# Association between Aspirin Therapy and Clinical Outcomes in Patients with Non-Obstructive Coronary Artery Disease: A Cohort Study

**DOI:** 10.1371/journal.pone.0129584

**Published:** 2015-06-02

**Authors:** In-Chang Hwang, Joo-Yeong Jeon, Younhee Kim, Hyue Mee Kim, Yeonyee E. Yoon, Seung-Pyo Lee, Hyung-Kwan Kim, Dae-Won Sohn, Jidong Sung, Yong-Jin Kim

**Affiliations:** 1 Department of Internal Medicine, Seoul National University College of Medicine, Seoul, Republic of Korea; 2 Cardiovascular Center, Seoul National University Hospital, Seoul, Republic of Korea; 3 Department of Statistics, Sungkyunkwan University, Seoul, Republic of Korea; 4 Institute of Health and Environment, School of Public Health, Seoul National University, Seoul, Republic of Korea; 5 Division of Cardiology, Department of Internal Medicine, Seoul National University Bundang Hospital, Seongnam-si, Gyeonggi-do, Republic of Korea; 6 Division of Cardiology, Department of Internal Medicine, Samsung Medical Center, Sungkyunkwan University School of Medicine, Seoul, Republic of Korea; 7 Center for Health Promotion, Samsung Medical Center, Sungkyunkwan University School of Medicine, Seoul, Republic of Korea; Medstar Washington Hospital Center, UNITED STATES

## Abstract

**Background:**

Presence of non-obstructive coronary artery disease (CAD) is associated with increased prescription of cardiovascular preventive medications including aspirin. However, the association between aspirin therapy with all-cause mortality and coronary revascularization in this population has not been investigated.

**Methods and Findings:**

Among the cohort of individuals who underwent coronary computed tomography angiography (CCTA) from 2007 to 2011, 8372 consecutive patients with non-obstructive CAD (1-49% stenosis) were identified. Patients with statin or aspirin prescription before CCTA, and those with history of revascularization before CCTA were excluded. We analyzed the differences of all-cause mortality and a composite of mortality and late coronary revascularization (>90 days after CCTA) between aspirin users (n=3751; 44.8%) and non-users. During a median of 828 (interquartile range 385–1,342) days of follow-up, 221 (2.6%) mortality cases and 295 (3.5%) cases of composite endpoint were observed. Annualized mortality rates were 0.97% in aspirin users versus 1.28% in non-users, and annualized rates of composite endpoint were 1.56% versus 1.48%, respectively. Aspirin therapy was associated with significantly lower risk of all-cause mortality (adjusted HR 0.649; 95% CI 0.492–0.857; p=0.0023), but not with the composite endpoint (adjusted HR 0.841; 95% CI 0.662–1.069; p=0.1577). Association between aspirin and lower all-cause mortality was limited to patients with age ≥65 years, diabetes, hypertension, decreased renal function, and higher levels of coronary artery calcium score, low-density lipoprotein cholesterol and high-sensitivity C-reactive protein.

**Conclusions:**

Among the patients with non-obstructive CAD documented by CCTA, aspirin is associated with lower all-cause mortality only in those with higher risk.

## Introduction

Coronary computed tomography angiography (CCTA) has been advocated as a useful diagnostic imaging test that provides anatomical evidence of coronary atherosclerosis. Especially among the patients with suspected coronary artery disease (CAD), CCTA could serve as a gate-keeper of downstream management including invasive coronary angiography or intervention.[1] With the increasing use of CCTA, more patients with non-obstructive CAD are being detected. Patients with non-obstructive CAD occupy 15%-30% of symptomatic subjects and 16% of asymptomatic subjects referred to CCTA.[2–4] Because non-obstructive CAD is frequently associated with the presence of vulnerable plaque, patients with non-obstructive CAD are at higher risk of mortality and cardiovascular events than those without.[2, 5–7] Despite the prevalence and the risk of non-obstructive CAD, appropriate management strategy is not established.

Several studies indicated that the detection of CAD by CCTA leads to more prescription of cardiovascular preventive therapies such as aspirin, statin, and anti-hypertensive medications.[8–10] Even without obstructive lesion that might cause myocardial ischemia, prescription of these preventive medications was significantly increased and intensified after CCTA in patients with non-obstructive CAD. The intensified statin and anti-hypertensive medications demonstrated improved cholesterol profile and blood pressure (BP), suggesting that the detection of non-obstructive CAD followed by increased preventive medications may lead the patients to better outcome.[9, 10] However, the effect of aspirin therapy in patients with non-obstructive CAD has not been established.[11–13]

In this study, we investigated the association between aspirin therapy and risk of all-cause mortality and a composite of mortality and coronary revascularization in patients with non-obstructive CAD (1–49% stenosis) documented by CCTA.

## Methods

### Ethics statement

This study followed the principles of the Declaration of Helsinki and was approved by the institutional review board of Seoul National University Hospital (H-1207-080-418). Because the records and information of study population were anonymized and de-identified for matching with the third party claims data, the requirement of informed consent for each individual was waived by the institutional review board.

### Study population

**Fig 1:**
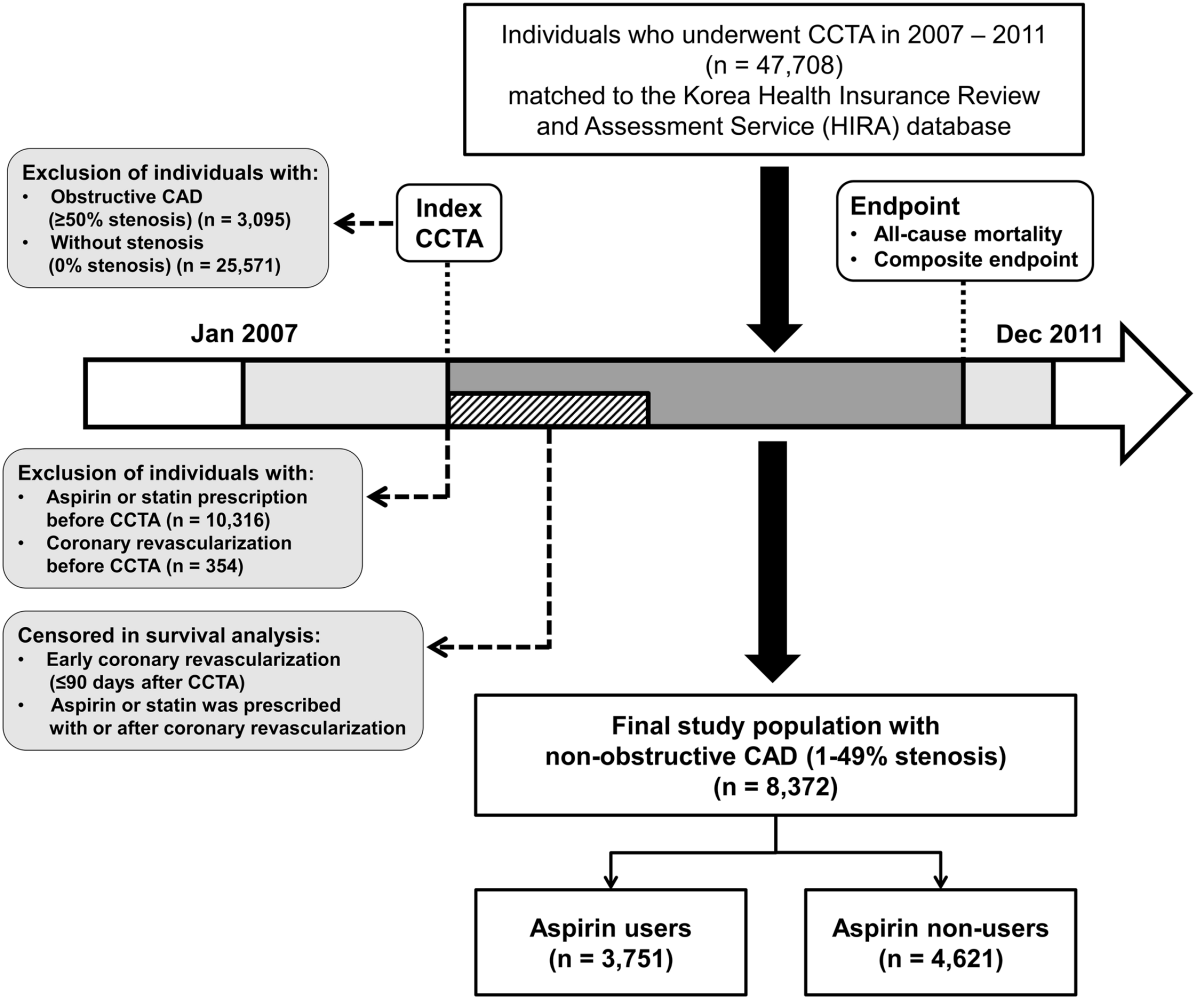
Selection of study population.

A detailed study protocol was published in our previous paper.[14] In brief, we identified a total of 8,372 consecutive patients with non-obstructive CAD (1–49% stenosis) among the cohort of 47,708 consecutive individuals who underwent CCTA at Seoul National University Hospital, Seoul National University Bundang Hospital, or Seoul National University Hospital Healthcare Gangnam Center from January 2007 to December 2011 (Fig 1). Patients for whom statin or aspirin was prescribed before CCTA (n = 10,316), and the patients who had prior coronary revascularization (n = 354) were excluded, to remove patients with existing coronary heart disease. We also excluded the patients with obstructive CAD (≥50% stenosis; n = 3,095) or normal CCTA results (0% stenosis; n = 25,571).

### Source of data

Using the electronic medical records, we obtained the resident registration numbers of study population with demographic factors and laboratory test results. The medical record information of study population was linked to the Health Insurance Review and Assessment Service (HIRA) claims data. Since HIRA has the universal coverage of the entire Korean population, it contains all information of medical service that was provided to each individual, including date, site, medications, diagnosis, procedures, hospitalization and survival.[14–17] Given that the novel antiplatelet agents such as ticagrelor and prasugrel were approved by Korean Food and Drug Association in 2013 after the study duration, information on these agents were not identified.

Personal information of study population was concealed, and an unidentifiable code was used for matching of the database. The matched data were kept securely at HIRA database. To confirm mortality cases and the accurate date of death, the HIRA data was cross-checked with the database from the Korean Ministry of Security and Public Administration.

### Outcome measures

The primary outcome measure was all-cause mortality during follow-up period, and the secondary outcome was a composite of all-cause mortality and late coronary revascularization (>90 days after CCTA; including percutaneous coronary intervention and coronary artery bypass graft operation). The date of initial CCTA was used as the index date to calculate the time to study outcomes, and follow-up duration of each patient was counted according to the first to event occurrence order. To minimize verification bias, patients who underwent early coronary revascularization (≤90 days after CCTA) and the patients for whom statin or aspirin was prescribed with or after coronary revascularization were treated as censored at the time of revascularization.[18]

### Coronary CT angiography

Patients underwent 64-slice multidetector CT (SOMATOM Sensation 64 and SOMATOM Definition, Siemens Medical Solutions, Forchheim, Germany; Brilliance 64, Philips Medical Systems, Best, The Netherland). For patients with a prescanning heart rate of 65–70 beats/minute or higher, 50–100 mg of oral metoprolol or 10–30 mg of intravenous esmolol was given 45–60 minutes prior to the CT examination, unless the patient had any contraindication to beta-blockers. During the image acquisition, 60–80 mL of contrast was injected, followed by a saline flush. CCTA was performed using the retrospective electrocardiography (ECG)-gated mode with ECG dose modulation, covering from the diaphragm to the level of tracheal bifurcation in caudocranial direction. The results of CCTA transferred to an external workstation and analyzed by 2 experienced radiologists independently. Coronary artery calcium scores (CACS) were measured using the previously described scoring system by *Agatston et al*.[19] The results of CCTA were classified as normal (0% stenosis), non-obstructive (1–49% stenosis) and obstructive (≥50% stenosis), according to the maximal stenosis of left main, left anterior descending, left circumflex, and right coronary arteries.

### Statistical analysis

Categorical variables were presented as frequencies with percentages, and continuous variables as means with standard deviations (SD) or medians with interquartile ranges (IQR). Group comparisons were performed by the use of Student’s independent t-test for continuous variables and the chi-square test for categorical variables. Survival analysis was performed using Kaplan–Meier method with log-rank test and Cox proportional hazard regression analysis for comparison of time to event outcomes. Univariable Cox proportional hazard regression analyses were performed to identify independent predictors of study outcomes, and all baseline characteristics and the data of medication prescriptions were considered as covariates for adjustment in multivariable Cox proportional hazard regression analyses. Data of laboratory tests were not entered into the multivariable regression model, because these parameters were incomplete in a small portion of the entire cohort. We performed subgroup analysis according to the age of 65 years, gender, and presence of diabetes or hypertension. For each subgroup, multivariable Cox proportional hazard regression analysis was performed using the baseline characteristics and medication prescriptions as covariates. Also, we added subgroup analysis according to CACS or results of laboratory tests among those each data was available: 7,488 patients for subgroup analysis by CACS; 7,015 patients by low-density lipoprotein cholesterol (LDL-C); 6,858 patients by high-sensitivity C-reactive protein (hsCRP); and 7,833 patients by glomerular filtration rate (GFR) calculated by the Modification of Diet in Renal Disease study equation. All statistical analyses were performed with software SAS 9.3 (SAS Institute Inc, Cary, NC, USA), and a P-value of <0.05 were considered statistically significant.

## Results

### Baseline characteristics

Demographic characteristics, the results of laboratory tests and CACS are summarized according to the use of aspirin (Table 1). Mean age of the total study population was 61.4 years (SD 10.9) and 70.3% were male. Aspirin was initiated after CCTA in 3751 (44.8%) patients. Compared to the aspirin non-users, aspirin users were older, with more frequent comorbidities and medication prescriptions, except the prevalence of liver cirrhosis and previous malignancies that were not different between the 2 groups. Of note, the proportion of patients with cerebrovascular disease was higher in the aspirin users (29.9% in aspirin users versus 13.8% in non-users; P <0.0001), and the proportion of patients on clopidogrel was also higher in the aspirin users (20.8% versus 6.7%; P <0.0001). Baseline fasting serum glucose and hemoglobin A1c were higher, and GFR was lower in aspirin-users. CACS was higher in the aspirin users.

**Table 1:** Baseline characteristics of total study population.

	Total population (n = 8372)	Aspirin non-user (n = 4621)	Aspirin user (n = 3751)	P value
**Age (years)**	61.4±10.9	59.1±10.9	64.2±10.2	<0.0001
**Male gender**	5886 (70.3%)	3313 (71.7%)	2573 (68.6%)	0.0020
**Diabetes mellitus**	1272 (15.2%)	416 (9.0%)	856 (22.82%)	<0.0001
**Hypertension**	2621 (31.3%)	904 (19.6%)	1717 (45.8%)	<0.0001
**Atrial fibrillation**	678 (8.3%)	285 (6.4%)	393 (10.5%)	<0.0001
**Heart failure**	470 (5.8%)	154 (3.5%)	316 (8.4%)	<0.0001
**Cerebrovascular disease**	1732 (21.2%)	609 (13.8%)	1123 (29.9%)	<0.0001
**COPD**	2043 (25.0%)	997 (22.5%)	1046 (27.9%)	<0.0001
**Liver cirrhosis**	145 (1.8%)	82 (1.9%)	63 (1.7%)	0.5572
Chronic kidney disease*	122 (1.5%)	47 (1.1%)	75 (2.0%)	0.0005
**History of malignancy**	860 (10.3%)	470 (10.2%)	390 (10.4%)	0.7345
**Medications**				
Statin	1983 (23.7%)	673 (14.6%)	1310 (34.9%)	<0.0001
Clopidogrel	1089 (13.0%)	309 (6.7%)	779 (20.8%)	<0.0001
ACEi	304 (3.6%)	53 (1.2%)	251 (6.7%)	<0.0001
ARB	1126 (13.5%)	362 (7.8%)	764 (20.4%)	<0.0001
Beta blocker	839 (10.0%)	270 (5.8%)	569 (15.2%)	<0.0001
CCB	780 (9.3%)	274 (5.9%)	506 (13.5%)	<0.0001
**Laboratory tests** †				
Hemoglobin (g/dL)	14.5±1.6	14.6±1.7	14.4±1.6	<0.0001
Total cholesterol (mg/dL)	194.2±41.3	196.2±39.7	191.6±42.9	<0.0001
Triglyceride (mg/dL)	137.1±87.2	134.1±88.3	140.7±85.7	0.0012
HDL-C (mg/dL)	50.4±12.5	50.8±12.5	50.0±12.5	0.0133
LDL-C (mg/dL)	116.6±30.3	118.4±29.6	114.3±31.0	<0.0001
Fasting glucose (mg/dL)	102.8±29.5	100.2±26.9	106.0±32.1	<0.0001
Hemoglobin A1c (%)	6.0±1.0	5.9±0.9	6.2±1.1	<0.0001
hsCRP (mg/L)	0.6±2.3	0.6±2.4	0.6±2.2	0.9454
GFR (mL/min/1.73m2)	77.3±17.8	79.4±18.5	74.6±16.2	<0.0001
**CACS** †	94.1±221.5	67.5±163.0	128.8±276.3	<0.0001
**Primary outcome**				
All-cause mortality	221 (2.6%)	123 (2.7%)	98 (2.6%)	0.8891
Follow-up duration	828 (385–1342)	680 (289–1220)	1021 (522–1443)	<0.0001
Annualized mortality rate (% person-year)	1.12	1.28	0.97	
**Secondary outcome**				
All-cause mortality or late coronary revascularization‡	295 (3.5%)	143 (3.0%)	152 (4.1%)	0.0070
Follow-up duration	802 (367–1335)	677 (288–1220)	984 (485–1429)	<0.0001
Annualized event rate (% person-year)	1.52	1.48	1.56	

Data are mean±SD, median (IQR; Q1–Q3) or number (%).

*Chronic kidney disease was defined as estimated glomerular filtration rate (GFR) <60 mL/min/1.73m^2^.

^†^Calculations of the laboratory tests and coronary artery calcium score were performed for those with available data of each component.

^‡^A composite of all-cause mortality and late coronary revascularization (>90 days after CCTA), including percutaneous coronary intervention and coronary artery bypass graft operation.

Abbreviations: COPD, chronic obstructive pulmonary disease; ACEi, angiogensin-converting enzyme inhibitor; ARB, angiotensin II receptor blocker; CCB, calcium channel blocker; HDL, high-density lipoprotein; LDL, low-density lipoprotein; hsCRP, high-sensitivity C-reactive protein; GFR, glomerular filtration rate; CACS, coronary artery calcium score; CCTA, coronary computed tomography angiography.

### Association between aspirin use and study outcomes

**Fig 2:**
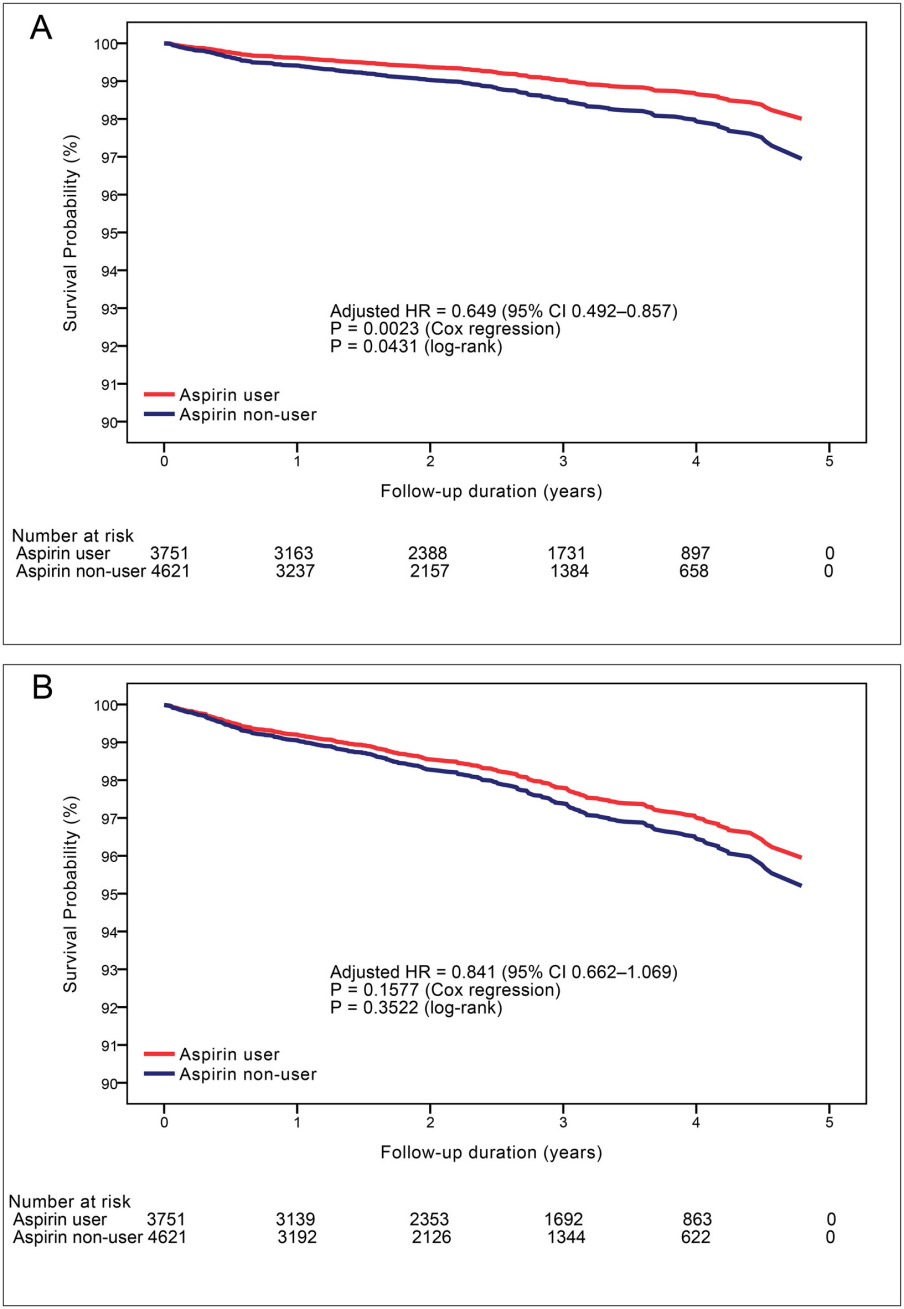
Risk-adjusted survival curves of aspirin users versus non-users. **A,** All-cause mortality-free survival by aspirin therapy in patients with non-obstructive coronary artery disease (1–49% stenosis). **B,** Composite endpoint (all-cause mortality or late coronary revascularization)-free survival by aspirin therapy. Survival analyses were performed using age, gender, comorbidities and concurrent medications as covariates.

During the 828 days of follow-up duration (IQR 385–1,342), 221 (2.6%) cases of all-cause mortality and 295 (3.5%) cases of the composite of all-cause mortality and late coronary revascularization were observed (Table 1). Annualized mortality rate was 0.97% in aspirin users and 1.28% in non-users. Multivariable Cox proportional hazard regression analysis showed that the use of aspirin after CCTA was significantly associated with lower risk of all-cause mortality (adjusted hazard ratio [HR] 0.649; 95% CI 0.492–0.857; P = 0.0023; Fig 2A and Table 2). For the composite endpoint, annualized event rate was 1.56% in aspirin users and 1.48% in non-users. In total study population, aspirin therapy was not associated with lower risk of the composite endpoint (adjusted HR 0.841; 95% CI 0.662–1.069; P = 0.1577; Fig 2B and Table 3).

**Table 2:** Cox proportional hazard model for all-cause mortality.

Variables	Unadjusted HR (95% CI)	P value	Adjusted HR (95% CI)	P value
Age (per 1 year)	1.127 (1.113–1.142)	<0.0001	1.130 (1.115–1.144)	<0.0001
Male gender	1.880 (1.136–3.112)	0.0140	1.328 (1.003–1.758)	0.0475
Diabetes mellitus	1.257 (0.921–1.715)	0.1501	1.094 (0.793–1.511)	0.5839
Hypertension	0.724 (0.455–1.100)	0.1242	0.674 (0.411–1.103)	0.1166
Statin	0.323 (0.215–0.483)	<0.0001	0.397 (0.262–0.602)	<0.0001
Aspirin	0.760 (0.583–0.992)	0.0437	0.649 (0.492–0.857)	0.0023
Clopidogrel	0.998 (0.653–1.527)	0.9931	0.984 (0.692–1.400)	0.9303
Beta blocker	1.264 (0.784–2.038)	0.3364	1.352 (0.827–2.210)	0.2292
CCB	0.651 (0.410–1.033)	0.0683	0.665 (0.392–1.130)	0.1317
ACEi	1.035 (0.564–1.899)	0.9122	1.077 (0.556–2.087)	0.8252
ARB	0.761 (0.522–1.109)	0.1550	0.924 (0.567–1.507)	0.7526

Variables in the model are as follows: age, gender, diabetes, hypertension, and the use of statin, aspirin, clopidogrel, beta blocker, CCB, ACEi, and ARB.

Abbreviations: ACEi, angiogensin-converting enzyme inhibitor; ARB, angiotensin II receptor blocker; CCB, calcium channel blocker; CI, confidence interval; HR, hazard ratio.

**Table 3:** Multivariable Cox proportional hazard model for the composite endpoint*.

Variables	Unadjusted HR (95% CI)	P value	Adjusted HR (95% CI)	P value
Age (per 1 year)	1.095 (1.083–1.107)	<0.0001	1.096 (1.084–1.109)	<0.0001
Male gender	1.895 (1.213–2.961)	0.0050	1.409 (1.098–1.807)	0.0070
Diabetes mellitus	1.502 (1.160–1.945)	0.0020	1.301 (0.995–1.701)	0.0546
Hypertension	0.931 (0.734–1.180)	0.5533	0.728 (0.456–1.164)	0.1853
Statin	0.426 (0.310–0.586)	<0.0001	0.430 (0.310–0.597)	<0.0001
Aspirin	1.049 (0.834–1.319)	0.6808	0.841 (0.662–1.069)	0.1577
Clopidogrel	1.405 (0.995–1.986)	0.0537	1.200 (0.896–1.608)	0.2214
Beta blocker	1.123 (0.628–2.010)	0.6947	1.235 (0.767–1.988)	0.3853
CCB	0.747 (0.511–1.092)	0.1318	0.722 (0.472–1.104)	0.1325
ACEi	1.480 (0.929–2.358)	0.0991	1.451 (0.876–2.403)	0.1488
ARB	0.885 (0.649–1.207)	0.4390	1.028 (0.696–1.519)	0.8893

* Composite endpoint: a composite of all-cause mortality and late coronary revascularization.

Abbreviations: ACEi, angiogensin-converting enzyme inhibitor; ARB, angiotensin II receptor blocker; CCB, calcium channel blocker; CI, confidence interval; HR, hazard ratio.

### Subgroup analyses

**Fig 3:**
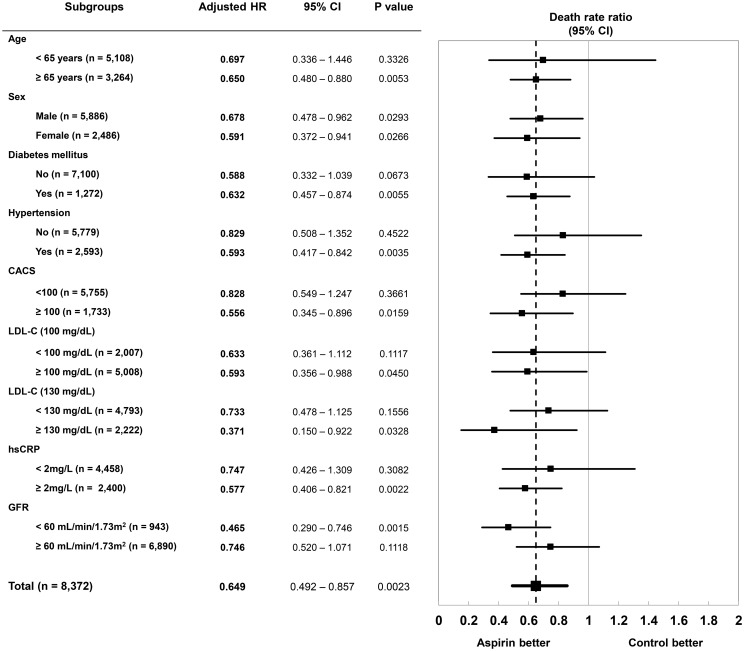
Association between post-CCTA aspirin therapy and all-cause mortality in subgroups. Risk-adjusted effects of aspirin therapy on all-cause mortality were analyzed in subgroups divided by age of 65 years, gender, presence of diabetes mellitus, presence of hypertension, and the results of CACS, LDL-C, hsCRP and GFR.

**Fig 4:**
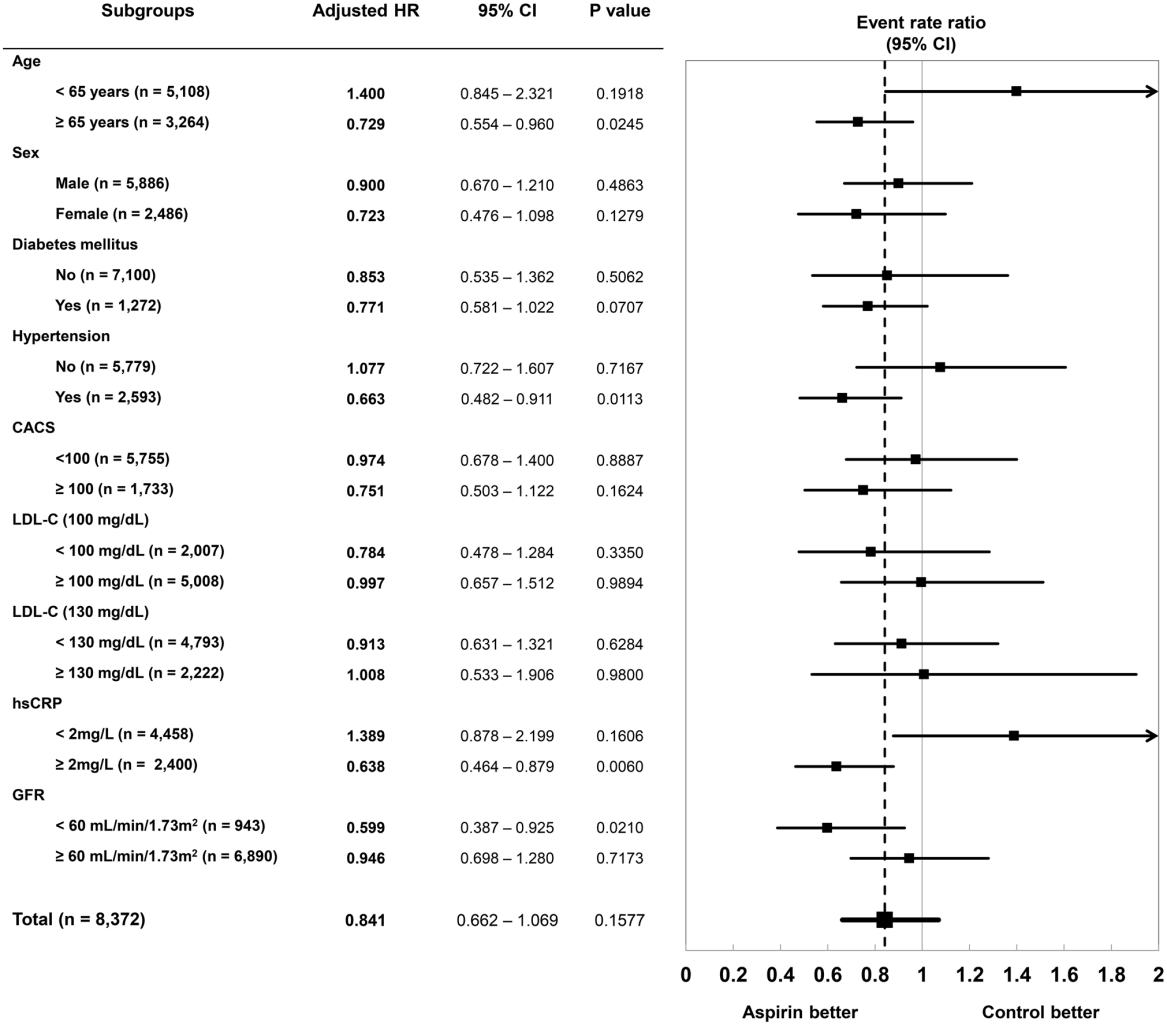
Association between post-CCTA aspirin therapy and the composite endpoint in subgroups. Risk-adjusted effects of aspirin therapy on the composite of mortality and late coronary revascularization (>90 days after CCTA) were analyzed in subgroups divided by age of 65 years, gender, presence of diabetes mellitus, presence of hypertension, and the results of CACS, LDL-C, hsCRP and GFR.

Although aspirin therapy was associated with lower risk of all-cause mortality, the effects were not consistent among the dichotomous subgroups (Figs 3 and 4). Patients with age ≥65 years, diabetes, hypertension, CACS ≥100, LDL-C ≥100 or ≥130 mg/dL, hsCRP ≥2 mg/L, or GFR <60 mL/min/1.73m^2^ showed significant association between aspirin therapy and lower risk of all-cause mortality, but the other subgroups did not. Similarly, overall beneficial effect of aspirin was not significant for the composite endpoint. However, prescription of aspirin after CCTA was significantly associated with lower risk of the composite endpoint among the patients with age ≥65 years, hypertension, higher hsCRP (≥2 mg/L) and lower GFR (<60 mL/min/1.73m^2^), and also the diabetic patients with a trend for a lower risk of composite endpoint.

## Discussion

We investigated the association between post-CCTA aspirin therapy and the risk of all-cause mortality and a composite of mortality and late coronary revascularization in 8,372 consecutive patients with non-obstructive CAD. Better clinical outcomes in aspirin users were observed in those with higher risk; the patients with age ≥65 years, diabetes, hypertension, decreased renal function, or higher CACS, LDL-C or hsCRP.

Currently, the use of CCTA is widely advocated not only for the detection of CAD in suspected patients but also as a reliable prognosticator and a gatekeeper for further management.[1, 20, 21] CCTA provides accurate detection of coronary atherosclerosis even in non-obstructive stage,[22] and more patients are being diagnosed as having non-obstructive CAD with the increasing use of CCTA.[2, 3, 5, 7] Because the presence of non-obstructive CAD indicates higher risk of mortality and cardiovascular events,[2, 5–7] the management strategy in this population is of clinical importance.

Detection of non-obstructive CAD is associated with more prescriptions of cardiovascular preventive medications. [8–10] According to a study by *McEvoy et al*, coronary atherosclerosis detected by CCTA resulted in the increased prescription of aspirin (odds ratio [OR]; 6.8 at 90 days and 4.2 at 18 months after CCTA) and statin (OR; 4.6 at 90 days and 3.3 at 18 months after CCTA), however, the increased prescription of statin and aspirin did not reduce cardiac events.[8] Still without results on clinical outcome, *Cheezum et al* reported that the use of aspirin and statin was increased upon the detection of non-obstructive CAD (OR; 6.9 for aspirin and 6.6 for statin), followed by reductions in total cholesterol and LDL-C.[9] More recently, *Hulten and Bittencourt et al* showed the intensified preventive medical therapies with significant improvements in lipid profile, and suggested that post-CCTA statin therapy may reduce cardiovascular events in the patients with non-obstructive CAD,[10] which was further clarified in our previous study.[14] However, there is no evidence regarding the use of aspirin in this population, which might be attributable to the lack of appropriate surrogate marker for aspirin therapy. Also, the use of aspirin confers increase in major bleeding and the net benefit of aspirin needs to be weighed between the bleeding risk and cardiovascular preventive effect, especially in patients with low risk.

Of note, the effect of aspirin for primary prevention is under debate and current guidelines advocate conflicting recommendations, because of the relatively lower event rates in the primary preventive setting and the unavoidable risk of major bleeding.[13, 23–26] Recent meta-analyses showed the benefit of aspirin on prevention of non-fatal myocardial infarction (MI), but reductions in cardiovascular mortality or all-cause mortality were not observed, and the risk of hemorrhagic stroke or gastrointestinal bleeding was significant.[27–29] Moreover, the Japanese Primary Prevention Project (JPPP) that assessed the effect of aspirin in patients with atherosclerotic risk factors was terminated early based on a futility assessment.[30] These results indicate that the use of aspirin for primary prevention should be individualized.[23, 31]

However, the benefit of aspirin for primary prevention is obvious in certain population. A recent report from the Multi-Ethnic Study of Atherosclerosis (MESA) demonstrated the potential of CAC measurement to guide aspirin therapy for primary prevention in low risk individuals, showing the ratio of benefit on reduction of coronary heart disease versus the risk of major bleeding was favorable in those with CACS ≥100 but not in those with zero CAC.[32] That study emphasized the net benefit of aspirin against the bleeding risk is higher in those with higher cardiovascular risk, and also implicated the clinical application of CACS to guide the use of aspirin.[31] Long term follow-up data of the Women’s Health Study also indicated that the risk of major bleeding increases with age, but the net benefit of aspirin for CVD risk is also greater at higher age.[33]

In this study, association between the use of aspirin and lower risk of mortality was observed only in subgroups with age ≥65 years, diabetes, hypertension, CACS ≥100, LDL-C ≥100 or 130 mg/dL, hsCRP ≥2 mg/L, or GFR <60 ml/min/1.73m^2^. These findings suggest that aspirin therapy in patients with non-obstructive CAD is beneficial only when they are at higher risk,[34–36] which is concordant with previous studies.[37] Given the consistent benefit of aspirin on non-fatal MI as shown in previous trials,[13, 27–29] it might be reasonable to use aspirin in primary prevention for patients with higher cardiovascular risk and with evidence of coronary atherosclerosis. On the other hand, the use of aspirin in patients with non-obstructive CAD is not justifiable among those with lower risk. For these lower risk patients, the absolute benefit from aspirin therapy would be far less than those with higher risk, while the risk of bleeding outweighs the net benefit.[13] Given the increased use of preventive medical therapies upon the detection of abnormal CCTA findings without evidence in light of clinical outcomes,[8–10] our results could be applied to not only the selection of patients for aspirin therapy after CCTA, but also the prevention against unwarranted aspirin prescription as well as potential bleeding risk.

The patients with cerebrovascular disease and the patients on clopidogrel were more frequent in aspirin users. According to the major clinical guidelines that were available before or during our study period, clopidogrel monotherapy was an acceptable option for secondary prevention of ischemic stroke, and the addition of aspirin to clopidogrel was not routinely recommended for patients with ischemic stroke or transient ischemic attack because of the risk of hemorrhage, unless they have a specific indication such as coronary stent or acute coronary syndrome.[38–41] In this study, the patients for whom aspirin was prescribed before the index CCTA and the patients who had prior coronary revascularization were excluded, and the patients for whom aspirin was prescribed with or after coronary revascularization were treated as censored at the time of revascularization. Therefore, the “clopidogrel users” would mainly indicate the patients with cerebrovascular events for whom dual antiplatelet therapy was initiated after the detection of non-obstructive CAD by CCTA. Regarding the combination of aspirin and clopidogrel for secondary prevention of stroke, previous trials demonstrated no significant benefit,[42, 43] and moreover, showed higher all-cause mortality because of the increased bleeding risk.[44] Because our study mainly focused on the all-cause mortality where the risk of fatal hemorrhagic event was reflected, the inclusion of the patients with cerebrovascular disease and those on clopidogrel could enhance the practical relevance and facilitate the further studies.

### Limitations

Interpretation of our results needs caution, given the following limitations. First, we could not provide information on symptomatic status, which might affect the event rates of study endpoints. However, we investigated the associations between aspirin therapy and clinical outcomes in primary preventive setting through exclusion of the patients with prior history of coronary revascularization, or prescription of statin or aspirin, the patients who underwent early coronary revascularization, and those for whom statin or aspirin was prescribed with or after coronary revascularization. Together with the selection of homogenous patients with non-obstructive CAD, our results provide hypothesis-generating evidence in real-world practice. Second, cardiovascular risk estimation was not available. Although we selected homogenous patients with non-obstructive CAD and it has been suggested that the presence of coronary atherosclerosis detected by CCTA might be discordant with the estimated cardiovascular risk,[45] the lack of 10-year risk scores limits the generalization of our study in part. Third, we could not provide data on any occurrence of major bleeding event, and therefore, quantitative comparison between the preventive effect and risk of bleeding by aspirin therapy was not possible. Because our study included the patients for whom the combination of aspirin and clopidogrel was used, our results should be interpreted with caution. However, we adopted the all-cause mortality as the main study outcome in which the fatal events from coronary heart disease or major bleeding would have been reflected. And it should be noted that, although the patients with dual antiplatelet therapy were included in the “aspirin users”, there was no reflection on the all-cause mortality, respectively the risk of bleeding. Fourth, certain information on the medications was not available, such as the compliance to aspirin therapy, the dosage of aspirin, and the use of anticoagulation. Given the recommendations from the guidelines that were available during the study period,[38–41] and the protocol of our study that excluded the patients for whom aspirin was prescribed before the index CCTA, the majority of aspirin users would have been prescribed for low dose aspirin. However, we recognize that the specific roles of aspirin therapy among the patients with coronary atherosclerosis should be further clarified.

## Conclusions

Among the patients with non-obstructive CAD (1–49% stenosis) documented by CCTA, aspirin therapy was associated with lower risk of all-cause mortality only in those with age ≥65 years, diabetes, hypertension, CACS ≥100, LDL-C ≥100 or 130 mg/dL, hsCRP ≥2 mg/L, and GFR <60 ml/min/1.73m^2^.

## References

[1] Shaw LJ, Hausleiter J, Achenbach S, Al-Mallah M, Berman DS, Budoff MJ, et al Coronary computed tomographic angiography as a gatekeeper to invasive diagnostic and surgical procedures: results from the multicenter CONFIRM (Coronary CT Angiography Evaluation for Clinical Outcomes: an International Multicenter) registry. Journal of the American College of Cardiology. 2012;60(20):2103–14. 10.1016/j.jacc.2012.05.062 . 23083780

[2] Min JK, Dunning A, Lin FY, Achenbach S, Al-Mallah M, Budoff MJ, et al Age- and sex-related differences in all-cause mortality risk based on coronary computed tomography angiography findings results from the International Multicenter CONFIRM (Coronary CT Angiography Evaluation for Clinical Outcomes: An International Multicenter Registry) of 23,854 patients without known coronary artery disease. Journal of the American College of Cardiology. 2011;58(8):849–60. 10.1016/j.jacc.2011.02.074 . 21835321

[3] Hoffmann U, Bamberg F, Chae CU, Nichols JH, Rogers IS, Seneviratne SK, et al Coronary computed tomography angiography for early triage of patients with acute chest pain: the ROMICAT (Rule Out Myocardial Infarction using Computer Assisted Tomography) trial. Journal of the American College of Cardiology. 2009;53(18):1642–50. Epub 2009/05/02. doi: S0735-1097(09)00541-5 [pii] 10.1016/j.jacc.2009.01.052 19406338 PMC2747766

[4] Choi EK, Choi SI, Rivera JJ, Nasir K, Chang SA, Chun EJ, et al Coronary computed tomography angiography as a screening tool for the detection of occult coronary artery disease in asymptomatic individuals. Journal of the American College of Cardiology. 2008;52(5):357–65. 10.1016/j.jacc.2008.02.086 . 18652943

[5] Jespersen L, Hvelplund A, Abildstrom SZ, Pedersen F, Galatius S, Madsen JK, et al Stable angina pectoris with no obstructive coronary artery disease is associated with increased risks of major adverse cardiovascular events. European heart journal. 2012;33(6):734–44. 10.1093/eurheartj/ehr331 . 21911339

[6] Hulten EA, Carbonaro S, Petrillo SP, Mitchell JD, Villines TC. Prognostic value of cardiac computed tomography angiography: a systematic review and meta-analysis. Journal of the American College of Cardiology. 2011;57(10):1237–47. 10.1016/j.jacc.2010.10.011 . 21145688

[7] Maddox TM, Stanislawski MA, Grunwald GK, Bradley SM, Ho PM, Tsai TT, et al Nonobstructive coronary artery disease and risk of myocardial infarction. Jama. 2014;312(17):1754–63. Epub 2014/11/05. 10.1001/jama.2014.14681 . 25369489 PMC4893304

[8] McEvoy JW, Blaha MJ, Nasir K, Yoon YE, Choi EK, Cho IS, et al Impact of coronary computed tomographic angiography results on patient and physician behavior in a low-risk population. Arch Intern Med. 2011;171(14):1260–8. 10.1001/archinternmed.2011.204 . 21606093

[9] Cheezum MK, Hulten EA, Smith RM, Taylor AJ, Kircher J, Surry L, et al Changes in preventive medical therapies and CV risk factors after CT angiography. JACC Cardiovascular imaging. 2013;6(5):574–81. Epub 2013/04/16. 10.1016/j.jcmg.2012.11.016 . 23582355

[10] Hulten E, Bittencourt MS, Singh A, O'Leary D, Christman MP, Osmani W, et al Coronary artery disease detected by coronary computed tomographic angiography is associated with intensification of preventive medical therapy and lower low-density lipoprotein cholesterol. Circ Cardiovasc Imaging. 2014;7(4):629–38. Epub 2014/06/08. 10.1161/CIRCIMAGING.113.001564 . 24906356

[11] Collaborative overview of randomised trials of antiplatelet therapy--I: Prevention of death, myocardial infarction, and stroke by prolonged antiplatelet therapy in various categories of patients. Antiplatelet Trialists' Collaboration. BMJ. 1994;308(6921):81–106. 8298418 PMC2539220

[12] Antithrombotic Trialists C. Collaborative meta-analysis of randomised trials of antiplatelet therapy for prevention of death, myocardial infarction, and stroke in high risk patients. BMJ. 2002;324(7329):71–86. 11786451 10.1136/bmj.324.7329.71 PMC64503

[13] Antithrombotic Trialists C, Baigent C, Blackwell L, Collins R, Emberson J, Godwin J, et al Aspirin in the primary and secondary prevention of vascular disease: collaborative meta-analysis of individual participant data from randomised trials. Lancet. 2009;373(9678):1849–60. 10.1016/S0140-6736(09)60503-1 19482214 PMC2715005

[14] Hwang IC, Jeon JY, Kim Y, Kim HM, Yoon YE, Lee SP, et al Statin therapy is associated with lower all-cause mortality in patients with non-obstructive coronary artery disease. Atherosclerosis. 2015;239(2):335–42. Epub 2015/02/16. 10.1016/j.atherosclerosis.2015.01.036 . 25682032

[15] Kwon S. Payment system reform for health care providers in Korea. Health Policy Plan. 2003;18(1):84–92. . 12582111 10.1093/heapol/18.1.84

[16] Lee CH, Kim K, Hyun MK, Jang EJ, Lee NR, Yim JJ. Use of inhaled corticosteroids and the risk of tuberculosis. Thorax. 2013;68(12):1105–13. Epub 2013/06/12. 10.1136/thoraxjnl-2012-203175 . 23749841

[17] Kim L, Kim JA, Kim S. A guide for the utilization of Health Insurance Review and Assessment Service National Patient Samples. Epidemiology and health. 2014;36:e2014008 Epub 2014/08/01. 10.4178/epih/e2014008 25078381 PMC4151963

[18] Hulten E, Bittencourt MS, Ghoshhajra B, O'Leary D, Christman MP, Blaha MJ, et al Incremental prognostic value of coronary artery calcium score versus CT angiography among symptomatic patients without known coronary artery disease. Atherosclerosis. 2014;233(1):190–5. Epub 2014/02/18. 10.1016/j.atherosclerosis.2013.12.029 . 24529143 PMC5536845

[19] Agatston AS, Janowitz WR, Hildner FJ, Zusmer NR, Viamonte M Jr, Detrano R. Quantification of coronary artery calcium using ultrafast computed tomography. Journal of the American College of Cardiology. 1990;15(4):827–32. . 2407762 10.1016/0735-1097(90)90282-t

[20] Miller JM, Rochitte CE, Dewey M, Arbab-Zadeh A, Niinuma H, Gottlieb I, et al Diagnostic performance of coronary angiography by 64-row CT. N Engl J Med. 2008;359(22):2324–36. 10.1056/NEJMoa0806576 . 19038879

[21] Min JK, Shaw LJ, Devereux RB, Okin PM, Weinsaft JW, Russo DJ, et al Prognostic value of multidetector coronary computed tomographic angiography for prediction of all-cause mortality. Journal of the American College of Cardiology. 2007;50(12):1161–70. 10.1016/j.jacc.2007.03.067 . 17868808

[22] Cheng V, Berman D, Rozanski A, Dunning A, Achenbach S, Al Khdair D, et al Performance of the traditional age, sex, and angina typicality-based approach for estimating pretest probability of angiographically significant coronary artery disease in patients undergoing coronary computed tomography angiography: results from the multinational coronary CT angiography evaluation for clinical outcomes: an international multicenter registry (CONFIRM). Circulation. 2011;124(22):2423–32. 10.1161/CIRCULATIONAHA.111.039255 22025600 PMC3240578

[23] Matthys F, De Backer T, De Backer G, Stichele RV. Review of guidelines on primary prevention of cardiovascular disease with aspirin: how much evidence is needed to turn a tanker? European journal of preventive cardiology. 2014;21(3):354–65. Epub 2013/04/24. 10.1177/2047487312472077 . 23610452

[24] Perk J, De Backer G, Gohlke H, Graham I, Reiner Z, Verschuren M, et al European Guidelines on cardiovascular disease prevention in clinical practice (version 2012). The Fifth Joint Task Force of the European Society of Cardiology and Other Societies on Cardiovascular Disease Prevention in Clinical Practice (constituted by representatives of nine societies and by invited experts). European heart journal. 2012;33(13):1635–701. 10.1093/eurheartj/ehs092 . 22555213

[25] Vandvik PO, Lincoff AM, Gore JM, Gutterman DD, Sonnenberg FA, Alonso-Coello P, et al Primary and secondary prevention of cardiovascular disease: Antithrombotic Therapy and Prevention of Thrombosis, 9th ed: American College of Chest Physicians Evidence-Based Clinical Practice Guidelines. Chest. 2012;141(2 Suppl):e637S–68S. Epub 2012/02/15. 10.1378/chest.11-2306 22315274 PMC3278064

[26] Pearson TA, Blair SN, Daniels SR, Eckel RH, Fair JM, Fortmann SP, et al AHA Guidelines for Primary Prevention of Cardiovascular Disease and Stroke: 2002 Update: Consensus Panel Guide to Comprehensive Risk Reduction for Adult Patients Without Coronary or Other Atherosclerotic Vascular Diseases. American Heart Association Science Advisory and Coordinating Committee. Circulation. 2002;106(3):388–91. . 12119259 10.1161/01.cir.0000020190.45892.75

[27] Seshasai SR, Wijesuriya S, Sivakumaran R, Nethercott S, Erqou S, Sattar N, et al Effect of aspirin on vascular and nonvascular outcomes: meta-analysis of randomized controlled trials. Arch Intern Med. 2012;172(3):209–16. Epub 2012/01/11. 10.1001/archinternmed.2011.628 . 22231610

[28] Bartolucci AA, Tendera M, Howard G. Meta-analysis of multiple primary prevention trials of cardiovascular events using aspirin. The American journal of cardiology. 2011;107(12):1796–801. Epub 2011/04/13. 10.1016/j.amjcard.2011.02.325 . 21481826

[29] Sutcliffe P, Connock M, Gurung T, Freeman K, Johnson S, Ngianga-Bakwin K, et al Aspirin in primary prevention of cardiovascular disease and cancer: a systematic review of the balance of evidence from reviews of randomized trials. PloS one. 2013;8(12):e81970 Epub 2013/12/18. 10.1371/journal.pone.0081970 24339983 PMC3855368

[30] Ikeda Y, Shimada K, Teramoto T, Uchiyama S, Yamazaki T, Oikawa S, et al Low-dose aspirin for primary prevention of cardiovascular events in Japanese patients 60 years or older with atherosclerotic risk factors: a randomized clinical trial. Jama. 2014;312(23):2510–20. Epub 2014/11/18. 10.1001/jama.2014.15690 . 25401325

[31] Hennekens CH, DeMets DL. Prevention: Aspirin in primary prevention needs individual judgements. Nat Rev Cardiol. 2014;11(8):438–40. Epub 2014/06/25. 10.1038/nrcardio.2014.88 . 24958081

[32] Miedema MD, Duprez DA, Misialek JR, Blaha MJ, Nasir K, Silverman MG, et al Use of coronary artery calcium testing to guide aspirin utilization for primary prevention: estimates from the multi-ethnic study of atherosclerosis. Circ Cardiovasc Qual Outcomes. 2014;7(3):453–60. Epub 2014/05/08. 10.1161/CIRCOUTCOMES.113.000690 . 24803472 PMC4412344

[33] van Kruijsdijk RC, Visseren FL, Ridker PM, Dorresteijn JA, Buring JE, van der Graaf Y, et al Individualised prediction of alternate-day aspirin treatment effects on the combined risk of cancer, cardiovascular disease and gastrointestinal bleeding in healthy women. Heart. 2014 Epub 2014/12/06. 10.1136/heartjnl-2014-306342 . 25475110 PMC4536552

[34] Blaha MJ, Budoff MJ, DeFilippis AP, Blankstein R, Rivera JJ, Agatston A, et al Associations between C-reactive protein, coronary artery calcium, and cardiovascular events: implications for the JUPITER population from MESA, a population-based cohort study. Lancet. 2011;378(9792):684–92. 10.1016/S0140-6736(11)60784-8 21856482 PMC3173039

[35] Emerging Risk Factors C, Kaptoge S, Di Angelantonio E, Pennells L, Wood AM, White IR, et al C-reactive protein, fibrinogen, and cardiovascular disease prediction. N Engl J Med. 2012;367(14):1310–20. 10.1056/NEJMoa1107477 . 23034020 PMC3714101

[36] Tonelli M, Muntner P, Lloyd A, Manns B, Klarenbach S, Pannu N, et al Risk of coronary events in people with chronic kidney disease compared with those with diabetes: a population-level cohort study. Lancet. 2012;380(9844):807–14. Epub 2012 Jun 19. 10.1016/S0140-6736(12)60572-8 PubMed Central PMCID: PMC22717317. 22717317

[37] Hayden M, Pignone M, Phillips C, Mulrow C. Aspirin for the primary prevention of cardiovascular events: a summary of the evidence for the U.S. Preventive Services Task Force. Annals of internal medicine. 2002;136(2):161–72. . 11790072 10.7326/0003-4819-136-2-200201150-00016

[38] Goldstein LB, Adams R, Alberts MJ, Appel LJ, Brass LM, Bushnell CD, et al Primary prevention of ischemic stroke: a guideline from the American Heart Association/American Stroke Association Stroke Council: cosponsored by the Atherosclerotic Peripheral Vascular Disease Interdisciplinary Working Group; Cardiovascular Nursing Council; Clinical Cardiology Council; Nutrition, Physical Activity, and Metabolism Council; and the Quality of Care and Outcomes Research Interdisciplinary Working Group. Circulation. 2006;113(24):e873–923. Epub 2006/06/21. 10.1161/01.STR.0000223048.70103.F1 . 16785347

[39] Adams RJ, Albers G, Alberts MJ, Benavente O, Furie K, Goldstein LB, et al Update to the AHA/ASA recommendations for the prevention of stroke in patients with stroke and transient ischemic attack. Stroke; a journal of cerebral circulation. 2008;39(5):1647–52. Epub 2008/03/07. 10.1161/STROKEAHA.107.189063 18322260 PMC4198335

[40] Furie KL, Kasner SE, Adams RJ, Albers GW, Bush RL, Fagan SC, et al Guidelines for the prevention of stroke in patients with stroke or transient ischemic attack: a guideline for healthcare professionals from the american heart association/american stroke association. Stroke; a journal of cerebral circulation. 2011;42(1):227–76. Epub 2010/10/23. 10.1161/STR.0b013e3181f7d043 . 20966421

[41] Guidelines for management of ischaemic stroke and transient ischaemic attack 2008. Cerebrovasc Dis. 2008;25(5):457–507. Epub 2008/05/15. 10.1159/000131083 . 18477843

[42] Diener HC, Bogousslavsky J, Brass LM, Cimminiello C, Csiba L, Kaste M, et al Aspirin and clopidogrel compared with clopidogrel alone after recent ischaemic stroke or transient ischaemic attack in high-risk patients (MATCH): randomised, double-blind, placebo-controlled trial. Lancet. 2004;364(9431):331–7. Epub 2004/07/28. 10.1016/S0140-6736(04)16721-4 . 15276392

[43] Bhatt DL, Fox KA, Hacke W, Berger PB, Black HR, Boden WE, et al Clopidogrel and aspirin versus aspirin alone for the prevention of atherothrombotic events. N Engl J Med. 2006;354(16):1706–17. 10.1056/NEJMoa060989 . 16531616

[44] Benavente OR, Hart RG, McClure LA, Szychowski JM, Coffey CS, Pearce LA. Effects of clopidogrel added to aspirin in patients with recent lacunar stroke. The New England journal of medicine. 2012;367(9):817–25. Epub 2012/08/31. 10.1056/NEJMoa1204133 22931315 PMC4067036

[45] Pen A, Yam Y, Chen L, Dennie C, McPherson R, Chow BJ. Discordance between Framingham Risk Score and atherosclerotic plaque burden. European heart journal. 2013;34(14):1075–82. 10.1093/eurheartj/ehs473 . 23303659

